# Refractive Surgery in Myopic Children

**DOI:** 10.3390/jcm13154311

**Published:** 2024-07-24

**Authors:** Beata Urban, Alina Bakunowicz-Łazarczyk

**Affiliations:** Department of Pediatric Ophthalmology and Strabismus, Medical University of Bialystok, Waszyngtona 17, 15-274 Bialystok, Poland; alina.bakunowicz-lazarczyk@umb.edu.pl

**Keywords:** myopia, children, corneal refractive surgery, intraocular refractive surgery

## Abstract

In this paper, we summarize the current knowledge on refractive surgery performed in the myopic pediatric population. We describe the main concerns about refractive surgery in myopic children and the indications for refractive surgery in this age group. We present a range of surgical procedures that are being used for the management of unilateral/bilateral myopia in children: corneal refractive surgery (PRK, LASEK, LASIK, FS-LASIK and SMILE) and intraocular refractive surgery (phakic intraocular lens implantation, refractive lens exchange or clear lens extraction), with both their advantages and drawbacks. We also describe the various complications and measures to prevent them.

## 1. Introduction

Myopia has been a subject of major international research for decades, with a focus on understanding the development of this condition and how it can be prevented or slowed down. Recent evidence suggests that the etiology of myopia can be attributed to a complex interaction of environmental exposure, genetic susceptibility and lifestyle factors, which leads to progressive axial elongation. A thorough knowledge of the cellular and molecular mechanisms is indispensable in the occurrence and development of myopia.

Myopia is the most common eye disease worldwide, affecting an estimated 2.6 billion people. It is projected that half of the world’s population will have some degree of myopia by 2050 [1]. The rising prevalence of myopia is also accompanied by an earlier onset, which consequently leads to an increased risk of high myopia. It is obvious that myopia reduces quality of life, limits occupational options, negatively affects the school life and mental health of children, and represents both a public health problem and a severe economic burden on society [2,3]. All these factors indicate how important it is to prevent myopia onset and to slow down its progression. 

Most children with low and moderate myopia can be successfully treated with prescription eyeglasses. Unfortunately, spectacles do not solve all aspects related to myopia. The problem arises when a child has high myopia (≥−6.0 diopters (D)). Failure with spectacles may be assigned to the limited field of view through eyeglass frames and prismatically induced optical aberrations. Even symmetric high myopia may result in visual distortions. Moreover, high minus lenses may also cause minification that limits potential best-corrected visual acuity (BCVA) [4]. It is also difficult not to mention the cosmetic and psychological issues or interference with sports and activities.

If there is a significant difference in refractive error between the two eyes, we are dealing with anisometropia. This, in turn, is the most frequent cause of amblyopia because corrective glasses will not provide the desired effect when the refractive difference between the eyes is greater than −2.00 D [5]. When eyeglasses are used in substantial anisomyopia, aniseikonia appears—a significant difference in the perceived size of the retinal image, which causes a loss of binocular vision and amblyopia in the eye with higher myopia [6]. Anisometropia greater than 6 D in myopic children is amblyogenic in 100% of them [5]. Conventional therapy of amblyopia (patching and/or pharmacologic and/or optical penalization of the sound eye) is usually effective in about two-thirds of the cases, but in a subset of children, this treatment is inadequate, with unacceptable vision despite adequate amblyopia therapy. Moreover, some children are resistant to wearing eyeglasses, and sometimes parents do not want their children to wear eyeglasses due to cosmetic or social concerns. Finally, children with developmental delay, autism, Down’s syndrome or cerebral palsy may not be able to wear eyeglasses.

Contact lenses have an advantage over spectacles in correcting myopia in children. They provide a larger visual field, equal retinal image size in both eyes and better quality of vision, especially in high myopia. However, contact lenses may not be a practical option. They have some disadvantages, such as difficulty with application and removing, foreign body sensation, being time-consuming for parents, frequent loss of contact lenses, poor compliance, intolerance to extended wear, risk of microbial keratitis and a relatively high cost [4,7,8,9,10]. Moreover, they may not be tolerated by many children, particularly those with neurobehavioral disorders [11].

According to Paysee, the risk factors for conventional therapy failure include a visual acuity of ≤20/200, astigmatism of >1.5 D, an age above 6 years, poor compliance and an insufficient understanding by parents [9]. For those myopic children who cannot wear spectacles or contact lenses, who have failed conventional therapy for anisomyopic amblyopia and who have behavioral or developmental problems, refractive surgery may be an alternative [12,13,14]. It should be performed as a last resort—when a child, especially with anisomyopia and the danger of amblyopia, is resistant to conventional therapy. The treatment goal here is to improve visual acuity, which can improve their social development and quality of life [15]. Yet, at the same time, we have to be aware that refractive surgery in children is still controversial for some surgeons and ophthalmologists.

## 2. Concerns about Refractive Surgery in Myopic Children

Refractive surgeries are surgical procedures used to correct refractive errors (such as myopia) by (A) reshaping the cornea to reduce its refractive power (corneal refractive surgery) or (B) by implanting an intraocular lens or refractive lens exchange (intraocular refractive surgery).

There are some specific challenges when it comes to laser refractive surgery for myopic children. One of the most relevant issues is the child’s young cornea. Corneal biomechanics in children have not been well studied and can change over time [16]. It is known that a younger cornea is less rigid when compared to an older one [9,16]. Increased corneal elasticity in some of the operated younger children may contribute later in life to the development of some form of fruste keratoconus that cannot be diagnosed at such an early age. Some of these young patients may be at risk of developing iatrogenic corneal ectasia in the future [17]. Moreover, the laser procedure modifies the shape of the cornea, and sometimes, it may be difficult to predict the stability of these changes in shape.

Another problem is associated with anesthesia. Nearly all laser procedures in myopic children are typically performed under general anesthesia. But it means that a child cannot fixate during the surgery. Moreover, specific precautions have to be taken before and during the procedure to prevent the unwanted effects of inhalational anesthetic agents on laser performance. For this purpose, special procedures have been developed in order to resolve this problem [18]. Moreover, the need for general anesthesia means the need for postanesthesia monitoring and dealing with postoperative pain.

Unclear refractive stability over time is another issue of laser refractive surgery in children. Anticipation of future eye growth must be considered when planning eye surgery in young children. The human eye reaches its adult size at approximately 14 years of age, and therefore, a child’s refraction is by no means stable because their eyes are still growing. In addition, axial length is regarded as the primary determinant of myopia [19]. Most (84.4%) of the myopic children in the COMET (The Correction of Myopia Evaluation Trial) cohort showed high rates of axial elongation during the first 3 years after baseline, followed by slower axial elongation between ages 13 and 16 years [19]. The average age of stabilization of the axial growth of 364 myopic children in COMET was 16.3 years. In 48 participants, axial length was still increasing till the age of 23. Changes in axial growth in young myopic children have a direct impact on their refractive errors and can affect the long-term outcomes of early laser refractive surgery. Refractive surgery, thereby, does not eliminate the need for the correction of residual refractive error with glasses or contact lenses, particularly for high myopia [4]. Moreover, children still need to continue amblyopia therapy because both the residual refractive error and amblyopia requiring treatment may be present after refractive surgery. Amblyopia therapy after laser procedure is especially indicated in children up to 6–7 years old (in older children, amblyopia is usually resistant to conventional treatment) [9,20]. When an older child is operated on, refractive surgery has a weaker impact on current amblyopia. In view of the fact that the child’s refractive error can change after refractive surgery, it is worth considering all future treatment options (corneal surface ablation procedures after PRK, pIOL exchange, piggyback IOL, and/or LASIK after previous IOL) [16].

At this point, it is also necessary to mention another issue, myopic regression, which usually occurs during the first year after laser surgery. Soon after starting corneal refractive surgery, it appears that refractive regression occurs after any laser procedure due to the tendency of the human eye to shift toward its original refraction after a period of desired refraction has been achieved postoperatively [21,22]. Refractive regression is defined as residual myopia (or hyperopia) of 0.25 D or a greater change occurring during follow-up, and it is caused by changes at the level of the cornea (not in axial length) [6,23]. The high rate of regression can be assigned to the enhanced healing properties of young eyes [12]. Myopic regression is one of the main complications in children, especially in very high myopia (>−12.0 D) [4,8,13,24,25]. In the pediatric population, it can be as high as 1.00 D per year [25]. It is not known when it slows down or stops. Based on his own experience, Tychsen estimates that it happens 5 to 8 years after refractive surgery [12]. To avoid this issue, it is suggested to overcorrect the operated eye and to achieve a target of slight hyperopia in myopia correction [16]. For myopic children, age 10 years or younger, 1–2 D overcorrection may be useful [12]. The application of drops with corticosteroids and the use of oral vitamin C for up to one year after surgery can be helpful in the inhibition of regression [26].

Another possible matter of some concern is the complications arising from the laser procedure itself. One of them, and probably the most frequently mentioned, is related to flap dislocation [4]. The main findings observed in adults were folds or microstriae, diffuse lamellar keratitis and epithelial ingrowth, including the partial splitting of the flap and a crack in the epithelium of the flap’s edge [27,28]. Flap dislocation was observed after trauma, and it is known that children are susceptible to injuries. There is always susceptibility to trauma in young patients, even years after corneal refractive surgery because children are active and they can be prone to eye injuries. Also, eye rubbing is a problem often encountered in the pediatric population.

Corneal haze is the next complication, which especially occurs in myopic children with very high myopia [4,8,9,25,29]. In one of the studies, corneal haze was observed in 22% of patients, especially in younger children [11]. Corneal haze is a manifestation of a pathological healing process; also, it is unpredictable in the pediatric population. Corneal haze represents subepithelial corneal fibrosis, and it appears as a result of an epithelial-stromal lesion involving a break in the epithelial barrier [30]. It is an inflammatory response that leads to a change where keratocytes mature into myofibroblasts, and the deposition of glycosaminoglycans and collagen in the anterior stroma during the healing period occurs, causing loss of corneal transparency [31]. With higher refractive corrections, a greater keratocyte response is pronounced, which leads to a more intense healing response [32]. Topical application of off-label mitomycin-C (MMC) is commonly used in adults at the end of their treatment to reduce haze through its mechanism of myofibroblast inhibition and reduction in keratocyte activity [33]. However, the long-term effects of MMC are not established, and for this reason, it is not universally used in pediatric refractive surgery. Topical corticosteroids and the use of systemic vitamin C can be helpful in reducing both corneal haze and related myopic regression [11].

Another challenge in both corneal and intraocular refractive surgery is the frequent lack of cooperation with children, which means that the postoperative care of a child is usually dissimilar to that of an adult patient. Sometimes, great difficulties occur during examination before/after surgery and in postoperative care (preventing manipulation of the eyes and application of medications, including necessary topical steroids). Despite the best efforts of parents, about 20% of special-needs children can be expected to dislodge their protective goggles and rub or poke their eyes [12]. Moreover, the surgeon has to be proficient in efficient examinations, especially since evaluation in a slit-lamp is often impossible.

Another problem associated with corneal laser procedures is dry eye. Dry eye disease (DED) is one of the most frequent complications after corneal laser refractive surgery, which is widely described in adults but rarely reported in the pediatric population [34,35]. Postoperative DED most commonly occurs after LASIK and FS-LASIK, while the incidence of dry eye after SMILE is lower and the recovery is faster (Table 1). DED is a multifactorial ocular disease with complex etiology. Postoperative dry eye is related to the transection of the corneal nerves, which impacts the cornea-blink reflex and tear production, resulting in neurotrophic epitheliopathy, reduced tear secretion, ocular surface inflammation, goblet cell damage and increased tear film instability [34,36]. Considering the fact that the prevalence of dry eye is higher in myopic patients, special attention should be paid to patients with high myopia who wear contact lenses for a long time if corneal refractive surgery is planned [37].

Because dry eye disease is rare in the pediatric population, its diagnosis is often overlooked, especially since children with mild ocular surface damage may report fewer dry eye symptoms than adults with similar changes [79,80]. Perhaps this is the reason why there are so few reported cases of pediatric dry eye after refractive surgery. Another explanation may be the fact that usually, short-term benefits are known, but late complications that are observed in adults may not be known at all in children [15]. Thus, further clinical long-term studies are essential to establish the prevalence of DED after refractive surgery in a pediatric population with many years to live. It is certainly worth subjecting to treatment with preservative-free lubricants before and after refractive surgery to facilitate visual rehabilitation and reduce the risk of secondary complications. Fortunately, most cases of dry eye resolve within the first year, but a small subset may lead to chronic dry eye [34].

## 3. When to Perform Refractive Surgery in Children

The minimum age of pediatric patients has not been established, so the best timing for performing refractive surgery is debatable. The youngest operated patient was 10 months old, and the results were good [43]. In the United States, more surgeons wait until the child is two years old before they begin to consider refractive surgery. This is understandable, given the fact that a child’s eye continues to grow during the first years of life and early childhood, leading to radical changes in eye refraction. Axial elongation and changes in corneal curvature are major factors influencing refractive changes in the first two years of life. The average corneal curvature flattens from 52 D at birth to 43.5 D at the age of 18 months, and the axial length increases from an average of 16.8 mm at birth to 23.6 mm in adulthood [81]. After 2 years of age, refraction is stabilized, and the results of refractive surgery are relatively stable and more predictable. Moreover, after 2 years of age, the cornea is less flexible. Therefore, children above 2 years of age have undergone successful procedures. The age of 10 years is often the upper age limit for performing refractive surgery, especially in amblyopic patients, where amblyopia is often preserved [9,20]. The best timing for performing refractive surgery is debatable; however, studies suggest that the best results are shown when the procedures are performed early [82]. On the other hand, there are studies concerning 7–16-year-old myopic patients that confirmed an improvement in their visual acuity in most eyes, and stereoacuity improved in half of the operated children [11,13,24,44,45,83,84,85,86,87]. This is also demonstrated in a meta-analysis of the research on pediatric refractive surgery in 213 anisometropic amblyopic eyes aged 1 to 17 years, which revealed that there was a significant overall visual acuity improvement after surgery [82]. Although it is true that amblyopia can be treated more effectively in younger children, many studies have shown that older children with amblyopia are also able to react to amblyopia treatment [88]. Interestingly, studies have shown that some amount of binocular cortical communication still exists in older amblyopes, which would explain the efficacy of amblyopia therapy beyond 7 years of age and at least until 15 years of age, suggesting that cortical plasticity is seen in late childhood [89]. Therefore, what is worth noting is that amblyopia treatment should be attempted in older children, and this also applies to refractive surgery.

## 4. Indications for Refractive Surgery in Children

Because many studies had, in the past, demonstrated excellent safety and effectiveness for refractive surgery in adults, laser refractive surgery in the pediatric population commenced in the 1990s [38]. The first procedures of photorefractive keratectomy (PRK) were reported in 1995, and laser-assisted in situ keratomileusis (LASIK) was reported four years later, in 1999 [38,47]. In turn, intraocular refractive surgery in children was first presented in 2000 [66]. Pediatric refractive surgery has been largely limited to off-label use, primarily because there are no studies that would analyze extremely long-term outcomes in refractive surgery-treated eyes. This especially concerns children who have potentially 70 to 80 more years to live [9].

Twenty years ago, O’Keefe and Nolan categorized the indications for pediatric refractive surgery as obligatory (children < 7 years of age with anisometropic amblyopia who do not accept spectacles/contact lenses), functional (older myopic children who participate in sports and other activities) and elective (older teenagers with indications for refractive surgery being the same as in adults) [20]. Currently, refractive procedures are carried out only on children with obligatory indications. The indications for pediatric refractive surgery are strictly limited to groups of the pediatric population who can especially benefit from these procedures where conventional therapies have failed. The first group includes children with severe anisometropia (usually 6 D) in cases where there is poor compliance with amblyopia therapy and a child is unable to tolerate or has failed in the traditional methods of correction using glasses or contact lenses [4,9,39]. The second group consists of children with high-magnitude anisometropia/myopia and concurrent neurobehavioral abnormalities associated with genetic mutations, ADHD, autism spectrum disorders, obsessive-compulsive disorder, Tourette’s syndrome, cerebral palsy or prematurity [13,39,67]. In these children, eyeglasses noncompliance or intolerance will lead to functional blindness in bilateral cases and thus, these patients are often operated bilaterally. The next group includes children with high myopia/anisomyopia with accompanying disorders, such as craniofacial abnormalities, ear deformities or neck hypotonia that make it impossible to use refractive correction [12,90]. Performing pediatric refractive surgery benefits the lives of both the child himself and his family, resulting from their improvement in visual acuity and child development—as the research has shown [40]. Other potential concerns include self-esteem, future career choice and interference with sports and activities. Functionally, it improves uncorrected and corrected distance visual acuity in myopic children and restores stereopsis and binocular fusion in many patients, with improvements in their behavior and environmental visual interaction [8,12,16,39,83].

## 5. Types of Refractive Surgery Performed in Myopic Children

Refractive surgery sees unprecedented growth; therefore, it is one of surgery’s fastest-growing areas. Refractive surgery encompasses all procedures that correct or minimize the refractive errors, among others, in myopic children. There are two types of surgery: (A) corneal refractive surgery, in which a laser removes the corneal tissue and reshapes the cornea to reduce its refractive power, and (B) intraocular refractive surgery, which is performed inside the eye, which consists of placing a phakic intraocular lens (IOL) implantation (to anterior or posterior chamber) or replacing the crystalline lens by means of refractive lens exchange.

Over the last few years, laser refractive surgery has been recognized as an extremely effective and safe procedure in adult patients, and at the same time, it has been proven to be a good treatment in children who have failed with traditional approaches. Photorefractive keratectomy (PRK), laser-assisted in situ keratomileusis (LASIK) with the flap created by either a mechanical microkeratome or femtosecond-based microkeratome (femtosecond laser-assisted in situ keratomileusis FS-LASIK), laser-assisted subepithelial keratomileusis (LASEK) and small-incision lenticule extraction (SMILE) have been used effectively to treat bilateral high myopia, anisomyopia and high astigmatic errors. These techniques are effective as well as safe for children and young adults who are not compliant with the conventional approaches. However, it is important to be aware that there is no golden standard or universally accepted refractive surgery technique for correcting myopia, and each procedure has its range of drawbacks and advantages.

The types of refractive surgery performed in myopic children with their briefly described techniques, main benefits and drawbacks are summarized in Table 1.

## 6. Laser Corneal Refractive Surgery

All surgical corrections are performed using an excimer laser (PRK, LASEK, LASIK) or femtosecond laser (FS-LASIK, SMILE). Although this technology is utilized in adults, some difficulties and challenges arise in finding proper handling with pediatric patients. Until now, laser corneal refractive surgery performed in myopic children can be divided into three categories: corneal surface ablation procedures (PRK, LASEK); corneal stromal ablation surgery, involving the creation of corneal flap (LASIK, FS-LASIK); and small-incision lenticule extraction (SMILE)—a form of stromal ablation that does not require a flap creation [2].

### 6.1. Photorefractive Keratectomy (PRK)

One of the first reports, from 1991, presented the use of an excimer laser for PRK in 31 sighted myopic eyes; patients under 18 years of age were excluded from the study (as in all subsequent studies on adults) [91]. Four years later, the first outcome of PRK in nine pediatric patients between 10 and 15 years was presented, with very promising results [38].

PRK was the first technique used for corneal surface ablation procedures with an excimer laser. In 1995, the FDA approved an excimer laser for use for surgical correction of refractive errors. Conventional PRK involves the removal of the corneal epithelium through mechanical debridement or dilute alcohol instillation or with a rotating brush. After removing the epithelium, computer-guided ablation of the underlying Bowman’s membrane and anterior corneal stroma is performed. In adults, the corneal epithelium heals in 3–6 days. In children, the time of complete epithelium healing is, on average, 3.5 days [11,39].

PRK is often a procedure of choice in the pediatric population due to a lesser risk of serious postoperative flap complications, including corneal flap dislocation or loss, striae, diffuse lamellar keratitis and increased risk of keratectasia [47,92,93]. Moreover, PRK has been shown to cause less biomechanical weakening of the cornea in comparison with other refractive procedures, so it can be performed on patients with high myopia because PRK leaves a thicker layer of untreated stroma for better corneal integrity [12]. Avoiding potential flap complications is especially important in children with neurodevelopmental disorders and cognitive disabilities [94]. On the other hand, the biggest downside to PRK is the higher rate of myopic regression due to the enhanced healing properties in young eyes, e.g., keratocyte-mediated regrowth and epithelial hyperplasia [11,12,94]. The average regression is about 0.6 D/year, so therefore, it cannot be ignored [11,12,14,22,29,41,87]. PRK is also associated with a higher risk of temporary or permanent corneal haze, reported in adults. Thankfully, corneal haze in a pediatric population treated with PRK is usually mild, mainly affects extremely high myopia patients and is effectively reduced by a postoperative topical steroid regimen, which inhibits the activation of fibrocytes [9,94]. Moreover, children neither demand nor expect immediate improvement in their vision [12]. Other disadvantages of PRK include immediate pain management and longer visual rehabilitation, but children handle discomfort better than adults [12]. Other drawbacks of PRK are associated with the imprecise removal of the corneal epithelium, which may result in asymmetric stromal hydration and delayed wound healing.

Although the procedure is still willingly performed in pediatric populations in order to avoid—among other things—the risks associated with flap creation in children, the indications for performing PRK have practically not changed for thirty years. The most frequent indication is high unilateral myopic amblyopia due to anisometropia of at least 6 D and contact lenses/spectacles intolerance [9,10,12,14,29,38,42,84,87,94,95,96]. Bilateral high myopia with neurodevelopmental disability is another indication [11,21,97]. Many surgeons prefer and consciously choose PRK because, in their opinion, PRK has a better risk profile for children, with fewer risks of serious postoperative complications [9,11,12,94]. They often perform PRK in the high myopic eye, with a mean preoperative spherical equivalent refractive error as high as −10 D to −13 D [9,10,14,29,42]. Currently, it is believed that PRK can often be used in situations where other procedures are contraindicated or to treat complications following other procedures [33]. The refractive goal for each child is emmetropia or the highest refractive error reduction that can be achieved while maintaining adequate treatment safety. This means the refractive treatment target is to decrease the anisometropia to 3.0 D or less [14,42,67]. In the opinion of Tychsen, in young myopic patients with myopia of >−6.0 D and thin corneas, phakic intraocular lens implantation (PIOL) can be a more suitable option [12].

Since 1995, the number of PRK procedures on myopic children has steadily grown, but most studies have involved a small number of myopic children (e.g., 5–6 patients in the 1990s and 11–35 patients in 2000–2010, respectively) [9,10,11,14,22,29,38,42,84,87,95,96]. Unfortunately, a common feature of all these examinations (except for a small number of operated children) was the short durations of follow-up—usually 12–24 months [9,10,22,29,42,87,96]. The first report on the long-term effects of PRK on the corneas of 10 myopic children is from 2019, and the follow-up was at least 5 years after surgery; however, at the same time, this study was limited by the small sample size (10 myopic eyes) [94]. The authors observed a stable reduction in refractive error; there was no visually significant corneal haze or corneal ectasia, so in their opinion, PRK was safe and effective over a 5-year follow-up period.

Despite the fact that myopic children were often older than 6 years of age and had severe amblyopia at presentation, visual acuity (uncorrected and best-spectacle corrected) and stereopsis improved in most operated eyes, even in older children, who were outside the standard age of visual plasticity [5,9,38,84,95,96]. Moreover, the visual acuity and binocular vision outcomes were significantly better in children who received permanent surgical correction of myopic anisometropia when compared to the control group of children who were treated conventionally with contact lenses [10,14]. A functional vision survey demonstrated a positive effect on the children’s ability to function in their environments after PRK, especially in children with neurodevelopmental disorders and cognitive disabilities [22,94]. The fact is, we still lack exceptionally long-term outcomes of treated eyes.

### 6.2. Laser-Assisted Subepithelial Keratomileusis (LASEK)

LASEK is considered a corneal surface ablation procedure and is a modified photorefractive keratectomy for low to moderate myopia, first introduced in 1999. The advantages of LASEK for adults include safety, economics, and excellent longer-term visual results; therefore, this procedure has gained worldwide acceptance when it comes to myopia correction.

Where in PRK, the epithelium is scraped off, in LASEK, a dilute solution of alcohol is applied for a short time, and a thin epithelial flap is created, so there is a more controlled removal of the epithelium. After excimer laser ablation, the flap is carefully repositioned over the ablated stroma immediately after surgery, which means faster visual recovery, less postoperative pain and faster epithelial healing when compared with PRK. Because LASEK is performed on the anterior cornea, the cornea’s biomechanical stability is maintained. The advantage of LASEK is the elimination of stromal flap complications, so it may be safer for children who are at an inherently higher risk of developing flap complications. The disadvantages include varying degrees of pain for 2 days and blurry vision for several days.

In one of the studies from 2001, comparable results between LASEK and PRK were found in adults with low or moderate myopia, and a further 63% of patients preferred the LASEK procedure. Soon after, it was performed on children [11,45,46]. The main indications for LASEK include myopic anisometropia greater than 4.00 D with amblyopia or high bilateral myopia and children noncompliant with spectacle or contact lens wear, often because of neurobehavioral disorders or prematurity [11,46]. Because LASEK is a flapless procedure, it is more suitable for patients with relatively thin corneas and high myopia, where PRK is associated with an increased risk of corneal haze and myopic regression. In such cases, LASEK could be a very good alternative with its superficial ablation and suppressed wound healing/haze, and this procedure has proven to be quite good in high myopic children, even in bilateral high myopia [44]. This has been confirmed by a study by Astle et al., who showed improvement in best-corrected visual acuity in 63.6% of myopic anisometropic children with amblyopia, and 87.95% had positive stereopsis 1 year after LASEK [43]. Another four studies analyzed the visual and refractive results of LASEK and PRK in myopic children, and all noted a reduction in the spherical equivalent postoperatively, as well as minimal corneal haze and improvement in binocularity; thus, both methods are effective and stable for correcting anisometropic myopia and bilateral high myopia in children who failed conventional methods of treatment [10,43,44,45]. Both procedures, PRK and LASEK, are recommended for adults with low and moderate myopia, but in children, these methods are often limited to off-label use when dealing with eyeglasses or contact lenses intolerance, so the treatment ranges for PRK and LASEK are very wide, from −2.25 D to −24.25 D [11,95].

### 6.3. Laser In Situ Keratomileusis (LASIK)

Improved surgical techniques resulted in the development of LASIK, which has become the most popular refractive surgical procedure. It was developed in 1990 by Lucio Buratto and Joannis Pallikaris as a combination of PRK and LASEK. LASIK quickly gained popularity due to its higher precision and reduced risk of complications compared with PRK and LASEK.

Laser in situ keratomileusis involves creating a central corneal flap composed of the epithelium, Bowman’s membrane and anterior stroma with mechanical microkeratomes. The flap is then reflected, and computer-guided excimer laser ablation of the posterior corneal stroma is performed. After changing the corneal stroma to correct the refractive error, the flap is repositioned again and sealed in place without sutures. LASIK, generally, is the procedure of choice for myopia to −11.0 D. There are several advantages of LASIK: faster visual recovery, less painful sensation, less corneal haze and maintenance of an intact Bowman’s membrane [15,57]. The main drawbacks concern the corneal flap, such as displacement, slip, dislocation, perforation, irregularity, and wrinkles and slip, as well as, less often, keratitis, epithelial ingrowth and iatrogenic keratectasia [57,98]. The limitations of this procedure have been proven in long-term studies related to the induction of aberrations and myopic regression [48]. One of the exclusion criteria is a corneal thickness value of less than 480 μm [24]. If the patient has myopia of >−8.0 D and a thin cornea, PRK is usually preferred [98].

LASIK has been performed infrequently on children. One of the first who conducted pioneering work on the use of LASIK to correct uniocular high myopia in children was Agarwal et al. [49]—a total of 16 eyes in 16 children aged 5–11 years received this surgery. The mean preoperative SE was −14.88 D, and the mean postoperative SE was −1.44 D. The authors concluded that utilizing LASIK for uniocular high myopia in pediatric eyes provided encouraging results where other measures failed. These results were the starting point for subsequent research, all involving children resistant to conventional treatment with spectacles/contact lenses with amblyogenic myopic anisometropia (very rarely, it was bilateral high myopia) [15,20,50,51]. The studies included a rather small number of unilateral myopic eyes (only in the study by O’Keefe and Nolan was there one child with bilateral high myopia), and all noted a reduction in the spherical equivalent postoperatively and better visual acuity after 1–2 years [20,49,50]. A notable disadvantage of all these reports, besides a small sample size, was a short follow-up period. The first retrospective study, published in 2022, involved 32 children aged 4–12 years with the longest follow-up time of 10 years after unilateral LASIK for myopic anisometropia of >6 D [24]. The mean preoperative SE was −10.3 D, and the mean postoperative SE was −1.3 D. Stability of the corneal refractive change was reached at 6 months in all eyes and remained stable for 10 years. BCVA improved from 0.04 ± 0.6 (in decimal notation) at baseline to 0.6 ± 0.2 after LASIK and occlusion therapy. Stereoacuity developed in 75% of children. Corneal clarity was stable, and there was no evidence of post-LASIK ectasia or any flap complications. All these results demonstrate that LASIK appears to be safe, effective and stable in selected groups of the pediatric population with high myopic amblyopia; however, further randomized studies with longer follow-up times, in which a larger group of children would participate, are essential.

### 6.4. Femtosecond Laser-Assisted *In Situ* Keratomileusis (FS-LASIK)

The femtosecond laser offers ophthalmic surgeons a novel tool for bladeless corneal incisions with high precision, and this laser is used in the initial phase of LASIK. For femtosecond laser-assisted in situ LASIK (FS-LASIK) surgery, a very thin corneal flap with a superior hinge is created using a femtosecond laser. After lifting the flap, the stromal bed is ablated using an excimer laser. Then, the residual stromal bed is washed with a balanced salt solution, and the flap is repositioned. FS-LASIK generates more reproducible and predictable flap diameters that have accurate thicknesses, and epithelial injury can also be decreased. The control and optimization of the corneal features may reduce flap-related complications such as reduced corneal nerve injury or dry eye.

The use of femtosecond laser in eye surgery began in the early part of the 21st century and has become increasingly popular in adults due to its greater safety when compared to LASIK. However, only a few reports on FS-LASIK in children appeared a few years later [52,85,86]. This procedure was performed successfully, among others, in high myopic amblyopic anisometropia in the pediatric population. There was an improvement in visual acuity, some children recovered near stereopsis, and no intraoperative or postoperative complications occurred in any patient, so the authors concluded that FS-LASIK could be a promising alternative method to these children who have failed success rates using traditional approaches [86]. However, further studies are required to determine the long-term stability and safety of the procedure in the pediatric population.

### 6.5. Small-Incision Lenticule Extraction (SMILE)

SMILE is a novel form of “flapless” surgery. In this procedure, first reported in 2008, an intracorneal lenticule is created using a femtosecond laser [99]. A small corneal incision is made by femtosecond laser, and then the lenticule is removed mechanically through a 3–4 mm corneal incision; thus, the same corneal tissue that is removed by the excimer laser in LASIK is eliminated in the SMILE procedure. In SMILE, there is no corneal flap; thus, many flap-related complications can be avoided. Another advantage of lenticular extraction over LASIK is the preservation of the anterior stroma, which is important for corneal biomechanics and maintaining corneal nerves [99,100]. SMILE induces a lower rate of keratocyte apoptosis, proliferation and inflammation compared with FS-LASIK [53]. This procedure is safe and effective in treating mild-to-moderate myopia, so the FDA approved the procedure in 2016 for the treatment of myopia less or equal to –10.00 D with or without astigmatism of a maximum of –5.00 D. Until now, around 3.5 million SMILE procedures have been performed worldwide in more than 70 countries [101]. Further studies have confirmed that SMILE appears to be safe, efficacious and highly predictable in the correction of myopia in adults, comparably with FS-LASIK [58]. Moreover, SMILE exhibited significant reductions in induced spherical aberrations compared with FS-LASIK [54]. Furthermore, dry eye symptoms and loss of corneal sensitivity in adults may occur less frequently after SMILE than FS-LASIK [55,58]. However, the conclusions should be interpreted with caution due to the low quality of data from cohorts and a short follow-up period [58]. In several reported studies, it was found that because SMILE does not disrupt the anterior corneal segments, it helps to achieve better postoperative corneal stability, so keratectasia following this procedure is rare [56,58].

The promising results of SMILE in adults encouraged surgeons to start performing this procedure in children with unilateral myopia. The main arguments for this treatment in pediatric populations include the decreased risk of iatrogenic ectasia and avoiding flap-related complications. Another important fact is that SMILE, being a tissue-saving procedure, has a positive effect on corneal nerve integrity [7,57]. Moreover, a number of studies have confirmed that the visual and refractive outcomes are similar to LASIK/FS-LASIK [54,56,58,98,102,103]. The indication for SMILE in pediatric populations is unilateral myopic anisometropic amblyopia resistant to conventional therapy [7,59,86]. In a small study by Zhang et al., visual acuity and contrast sensitivity improved in most of the operated children, some patients recovered near stereopsis, and no intraoperative or postoperative complications occurred in any patient; however, the time of observation was short (only 8 months after surgery) [86]. In a comparative case series study by Eissa et al., in 80% of operated children, visual acuity improved by 1–6 lines, and 20% gained 0–1 lines after SMILE; in more than 93% of patients, stereoacuity improved. No corneal ectasia was observed throughout the 18 months of follow-up; however, in two children, diffuse lamellar keratitis developed [7]. As suction loss is the most common complication of SMILE, the authors observed it in two of three patients after SMILE [7,104]. A large retrospective study by Samir et al., published in 2021, included 124 children with unilateral myopia who underwent SMILE [59]. During a 4-year follow-up, their visual acuity continued to improve. The authors mention that the intraoperative complications were scarce and relatively innocuous. Although no late complications were reported, the authors noted that a larger study with longer follow-up periods is necessary to determine the long-term effects of SMILE. This is important, considering that the study by Damgaard et al. showed that a minor group of adults with high myopic correction exhibited considerable refractive regression 7 years after SMILE [60].

## 7. Intraocular Refractive Surgery

### 7.1. Phakic Intraocular (Anterior or Posterior Chamber) Lens Implantation

This is an alternative procedure for the correction of high myopia, whereby a surgically implanted device is placed in front of the natural lens, which remains intact. Refractive surgery using phakic intraocular lens (pIOL) implantation is a reversible procedure. Phakic IOLs can be divided into 1 anterior chamber angle-supported pIOLs (withdrawn from the market in the early 2010s), 2 anterior chamber iris-fixated pIOLs, and 3 posterior chamber pIOLs placed in the posterior chamber behind the iris, fixated in the ciliary sulcus between the iris base and ciliary muscle. The FDA approved two types of pIOLs for adults: the anterior chamber pIOL called Verisyse (composed of PMMA) in the USA (in Europe, similar ones are Artisan made of PMMA and Artiflex made of polysiloxane), and the posterior pIOL called Visian Implantable Collamer Lens (ICL) [8]. Large numbers of studies have shown that pIOLs provide excellent optical outcomes regarding uncorrected visual acuity, distance-corrected visual acuity and residual refractive errors [105,106].

The first report of phakic intraocular lens implantation in children with high myopia dates back to 1999 [68]. Indications for implantation of pIOL in children are high anisomyopia or bilateral high myopia who are noncompliant with traditional therapy and high myopic amblyopia, which is associated with neurobehavioral disorders in children who cannot undergo corneal refractive surgery [66,69,70,71,72,107,108].

The main advantage of anterior chamber pIOLs is the increased distance between the artificial lens and natural lens so that the risk of developing cataracts is lower when compared to posterior chamber pIOLs. The disadvantages of anterior chamber pIOLs include progressive corneal endothelial cell loss and risk of traumatic iris-haptic de-enclavation due to eye rubbing [26,61,62,63]. To prevent eye rubbing, some children will need to be fitted with arm restraints for the first several postoperative weeks [12]. Cases of secondary glaucoma have also been reported. In a study conducted by Faron et al. in four eyes, pupillary block developed in the first 3 days after Visian IOL implantation, so surgical iridotomy was performed [26]. Tychsen et al. observed pupillary block in two children (8%) who underwent Visian IVL implantation [73].

Till the 2010s, there were only a few case reports on anterior chamber pIOL implantation in children, with a small number of patients and short follow-ups. The indication for surgery was myopic anisometropia of >−8.0 D in children who have been noncompliant with traditional medical treatment [26,61,62,63,64,65]. An iris-enclaved anterior chamber phakic intraocular lens (AC pIOL—Verisyse or Ophtec-Artisan) was implanted in all these studies. Another two studies involving a large group of patients have been published in the last three years. In a large case series, Faron et al. published the results of iris-enclave AC pIOL implantation in 115 eyes of 78 high myopic children aged 0.6–14.1 years, with a mean follow-up of 3.9 years [26]. Only children with an anterior chamber depth of ≥ 3.2 mm were included in the study. The visual results were very good, but it is worth pointing out the complications after the surgery. Retinal detachment occurred in four eyes (3%) at an average of 6 years after the procedure of pIOL implantation. An Ophtec-Artisan pIOL was explanted in six eyes. Four of these were the eyes that had cataract surgery due to a previous repair of a detached retina, one eye had a corneal ulcer after 3 years (caused by preexisting trigeminal hypesthesia) and the last eye had repeated traumatic de-enclavation of the pIOL. In nine eyes, the intraocular lens had to be repositioned due to trauma. However, according to the authors, the prevalence of major complications was relatively low, considering the fact that most of the operated children had visuomotor (86% of patients) and neurobehavioral (57% of patients) abnormalities. However, despite all this, the majority of children had an improved quality of life [26].

In a study conducted by Griščíková et al., 58 children with anisometropic myopia, aged 6.7 years on average, underwent anterior chamber iris-fixated pIOL implantation [63]. The mean follow-up was 38.5 months. The target refraction was emmetropia. The results showed that improved visual acuity and binocular vision quality were improved in 94.8% of the children. The mean preoperative endothelial cell count of 2874.7 cells/mm^2^ changed to 2685.3 cells/mm^2^ at 2 to 6 years postoperatively. All authors advocate larger prospective clinical trials with a much longer time of observation to assess the long-term effects of pIOLs.

In recent years, anterior chamber pIOL implantation has been gradually replaced by posterior chamber pIOL implantation. There are numerous advantages of using posterior chamber phakic IOL compared to corneal laser refractive surgery. First of all, the pIOL procedure is reversible, with the possibility to exchange the pIOL or add a piggyback lens in the ciliary sulcus to correct future errors. There is less risk of myopic regression over time, better contrast sensitivity, less high-order aberration, higher predictability, preservation of accommodation, quick visual rehabilitation, higher predictability and no complications related to laser surgery (flap complications, corneal haze, and/or corneal ectasia) [7,8,98]. The disadvantages include anterior subcapsular cataract formation, progressive endothelial cell loss, pigment dispersion and secondary glaucoma due to IOL capture or pupillary block [12,16,26,105].

With the appearance of a foldable, sulcus-positioned intraocular collamer lens, approved by the FDA in 2005 (Visian ICL) (Staar Surgical Co), this type of posterior chamber plano-concave lens became willingly chosen by surgeons for implantation in a pediatric population with high anisomyopia [12,26,66,71,73,75]. These lenses are implanted in children with myopia of up to around −17.00 D, who have a corneal diameter of >10.8 mm and anterior chamber depths of ≥3.2 mm [12]. 

Until recently, only a few case reports were available regarding the use of posterior chamber phakic IOLs in treating myopic amblyopia in children and young adults in the research literature [66,70]. Currently, with the introduction of new pIOL models, thanks to their beneficial properties, they are increasingly used in myopic pediatric populations [7,74,107]. Eissa et al. reported on 15 children with myopic anisometropia who underwent collamer lens Visian ICL/TICL) of the V4c design [7]. This fourth generation of collamer lenses is developed to provide more space between the pIOL and crystalline lens and prevent cataract formation. Moreover, the V4c model has an additional central hole of 0.36 mm to change the aqueous flow and eliminate the need for iridectomy. Nevertheless, there was one case of acute IOP elevation, which was resolved with a topical beta-blocker, and two cases of anterior subcapsular cataract. It is perhaps because of the position of the pIOL in proximity to the natural lens, which frequently induces friction; this can lead to cataract development and pigment dispersion [105]. The authors noted that endothelial cell loss did not change after 1–9 months, but it was statistically significant after 18 months [7]. Improvement in visual acuity by 3–6 lines was observed in 80% of the children. Morkos et al. noted similar results in 42 eyes of 42 children with myopic anisometropic amblyopia who underwent Visian ICL V4c implantation [74]. The results showed significant improvement in visual acuity and refractive stability. No complications were noted within the 14.7-month follow-up period. Long-term follow-up of possible late complications after a pediatric pIOL implantation is strongly encouraged. Interestingly, recent research has shown that ICL V4c implantation resulted in alterations in retinal and choroidal morphology (more pronounced in higher degrees of myopia) over a 1-year follow-up period [109].

Another new model of pIOL used recently in myopic children is the Eyecryl phakic IOL (Biotech Vision Care), which is a foldable, hydrophilic acrylic plate-haptic that is implanted in the sulcus and has a power range from –3.00 to –23.00 D. Morya et al. conducted a study on 23 eyes of 21 anisomyopic and isomyopic amblyopia children aged 10–19 years [107]. The mean intraocular lens power was −12.00 D. The results showed improvement in visual acuity and contrast sensitivity. The average endothelial loss recorded was 5.78% at 12 months after surgery, which is statistically insignificant. When it comes to glaucoma, IOP increased in only two eyes—at 1 day and 6 weeks. This may be due to the special construction of the lens, which has three holes: one in the optics and two in the peripheral haptics to facilitate the outflow of aqueous humour and thus prevent pupil block. There were no postoperative complications such as cataracts, dislocation of phakic IOL or retinal detachment.

### 7.2. Refractive Lens Exchange (RLE) or Clear Lens Extraction (CLE)

This procedure involves the removal of a natural crystalline lens with or without simultaneous insertion of a low-power or plano intraocular lens (IOL) that replaces or augments the refractive ability of the eye. With advances in cataract surgery and the introduction of new models of intraocular lenses, these techniques are being performed more commonly. Supracapsular (outside and above the capsular plane) microincision bimanual phacoemulsification is considered to be the preferred and safest approach [76]. RLE is a good option for developing countries due to its lower costs, as there is no need for a second surgery in the future (no development of cataracts) [76].

Proper patient selection is crucial for the success of RLE. It should be performed only in carefully selected children where laser corneal refractive surgery is not possible and in those who are unsuitable for phakic IOL correction. The main indications are severe anisomyopia and extremely high bilateral myopia (−12.0 D and above) in children noncompliant to spectacle or contact use, where corneal laser procedures are contraindicated, who also have a shallow anterior chamber of <3.2 mm and/or the corneal diameters too low and where phakic IOL is impossible or too risky to be implanted [110]. The main difference between standard cataract surgery and RLE (besides clear lens) is the presence of ocular abnormalities caused by high myopia, such as an unstable depth of the anterior chamber caused by decreased scleral rigidity.

A very important disadvantage of RLE is the loss of accommodation, which should be discussed thoroughly; therefore, it is crucial to inform young patients and their parents about it. This drawback can be mitigated by the implantation of intraocular lenses. Actually, when new premium types of IOLs (multifocal IOLs, accommodating IOLs, trifocal IOLs and extended-depth-of-focus (EDOF) IOLs) are introduced, concerns about loss of accommodation for young patients after RLE drastically reduce, and spectacle-free clear vision across all distances can be achieved [76].

Faron and Tychsen evaluated the safety and efficacy of RLE in children with unilateral/bilateral high myopia, with a mean follow-up of 6.4 years [75]. A total of 94 eyes in 57 children aged 1.5–22 years (mean 8.7 years) underwent surgery. A total of 83% of the children had strabismus and/or nystagmus. IOL power was chosen to achieve a target refraction of 0 to +1 D in all children. Average preoperative and postoperative refractions were −15.6 ± 4.4  D and −1.15 ± 1.2 D at the 5-year follow-up, respectively. Corrected and uncorrected distance visual acuity improved by 0.32 logMAR and 0.80 logMAR, respectively. A total of 13% of eyes required YAG laser capsulotomy. The authors concluded RLE can effectively improve visual acuity and quality of life in properly selected highly myopic children. Similar promising results were obtained in previous research on myopic children with neurobehavioral disorders who had RLE with/without IOL, with a follow-up of about 4 years [77,78]. Surgery was very effective in improving their functional vision, with myopic regression being −0.16 to −0.43 D/year. Reported complications included dislocation of IOL, hyphema and retinal detachment in one child after trauma (4%). It is essential to remember the postoperative complications of RLE. The most frequent complication is late aphakic/pseudophakic retinal detachment, with incidences from 1.5–2.2% to 8.1% [111,112]. Barrier diode laser may be applied to children’s eyes whose axial length exceeds 28.0 mm in order to avoid or reduce RD [78,110]. All authors agree that further studies are required, as serious complications may only appear after a prolonged follow-up; thus, further study is necessary to determine the long-term safety of RLE in pediatric populations.

## 8. Future of Refractive Surgery

In the last few years, refractive surgery has made rapid growth, and there is still much space for development and potential market demand. Currently, every company in the refractive surgery devices market is developing new platforms for both excimer and femtosecond laser technologies [113]. Looking ahead, future improvements in femtosecond laser technology would increase its use not only in corneal refractive surgery (flap creating, corneal incision and lenticule extraction) but also in intraocular procedures. Nowadays, the femtosecond laser is used in cataract surgery (clear corneal incisions, capsulorhexis, lens fragmentation and arcuate incisions), but still, femtosecond laser-assisted cataract surgery (FLACS) remains in its basic stages of development [114]. Femtosecond laser technology will be evolving to incorporate shorter pulse durations and the robotization of lens aspiration steps [115]. There is also a tendency towards implementing new equipment with a much smaller footprint, which is important due to the increasing number of refractive procedures moving to in-office surgery.

Big data and artificial intelligence (AI), which are increasingly applied in ophthalmology (e.g., for diabetic retinopathy), will be more widely used in refractive surgery. With improved computing power and the ability to collect, store and analyze big data, it is now possible to form a model to predict suitability for refractive surgery (LASIK, SMILE) or to predict laser refractive surgery outcomes and enhance the accuracy for SMILE outcomes [116]. There are already AI-based nomograms for SMILE, so it is quite possible that AI will be used in the development of pediatric nomograms, which are still lacking [117,118]. AI algorithms analyze patients’ eyes with remarkable detail, allowing for customized procedures tailored to individual corneal topography.

It can be anticipated that in the future, refractive surgery will no longer be limited to the procedures mentioned, as we can most likely expect some innovations in both laser vision correction (LVC) and lens-based refractive surgery.

### 8.1. Laser Vision Correction (LVC)

Laser corneal refractive surgery has been performed for over three decades. In recent years, extraordinary progress has been made with diagnostic methods and in the field of operational techniques using the latest technologies, which have made laser procedures safe, precise and predictable.

Standard corneal surface ablation procedures, over time, will be performed less frequently now that clinical applications of various new technologies will elevate refractive surgery to a new level. Nowadays, topography-guided LASIK and wavefront-guided LASIK technologies allow results that were not previously possible [113]. The newest laser procedure—SMILE—is currently the fastest-growing type of LVC on the medical market, among others, thanks to no flap complications and very fast recovery rates.

Looking into the future, the next step forward in LVC could be laser-induced refractive index change (LIRIC). This innovative technology can be applied to the cornea, and it affects the refractive index of the cornea rather than its shape [119]. In this method, a low-energy femtosecond laser is used to alter the corneal collagen structures to change the refractive index. LIRIC only treats up to a 10 μm depth of the cornea. It is a non-incisional, non-ablative technique; therefore, it could cause fewer side effects than traditional laser refractive surgeries. This technology will be commercially available in a few years, and it has the potential to displace LASIK, SMILE and PRK.

### 8.2. Lens-Based Refractive Surgery

RLE is often combined with simultaneous implantations of the intraocular lens (IOL). In recent years, the premium IOL market has expanded considerably. Many new models of lenses have been introduced and they are successfully implanted in adults, especially since the need for innovation in postoperative refractive adjustments is greater than ever. For example, one such lens is the trifocal IOL, which splits 25% of light for near, 25% for intermediate, and 50% for distance vision. The extended-depth-of-field IOL (EDOF) IOL is another example of a lens implanted in adults during RLE, and this lens can be easily removed compared with other IOLs. The light-adjustable intraocular lens (LAL) is another new model of IOL implanted during RLE, which is made of a material that will react to UV light and adjust the lens power after surgery, with excellent visual outcomes [120,121]. However, it should be noted that patients with extreme myopia are beyond the manufacturers’ power ranges for trifocal, EDOF and LAL lenses. It should be expected that the available power ranges and sizes of the IOL, however, will expand.

Modular IOL systems that allow for stable placement and easy replacement of existing IOLs are another new frontier. Notably, pediatric patients represent a challenging population, considering the growth of the eye postoperatively with the associated refractive changes. In these children, foldable modular IOL systems with separate base and optic components, allowing for safer and easier IOL exchange or removal, could be a good option in the future. Currently, trials are underway to evaluate the safety of the Harmoni modular intraocular lenses that have a separate base and optic components [122]. Moreover, scientists are conducting research on using the Juvene IOL—a new fluid-filled, modular, accommodating intraocular lens (IOL) system [123]. Nowadays, the number of office-based lens surgeries for refractive lens exchange or phakic intraocular lens implantation is increasing, and many patients will choose to undergo RLE instead of LVC [124]. The removability of the modular lens can make it more appealing than a refractive surgery for some patients.

In the pediatric population, refractive surgery has been largely limited to off-label use in cases of anisometropic amblyopia and bilateral high myopia with neurodevelopmental disability when a child refuses traditional therapies. If high-quality, prospective, randomized controlled trials with long follow-ups prove their effectiveness and safety, refractive surgery may become a part of standard practice in the decades ahead for special-needs children.

## Figures and Tables

**Table 1:** Summary information about main refractive procedures performed in myopic children.

Procedure	Method	Advantages	Disadvantages	References
Corneal refractive surgery				
PRK (photorefractive keratectomy)	Mechanical removal of corneal epithelium, ablation of the underlying Bowman’s membrane and anterior corneal stroma	Less biomechanical weakening of the cornea No stromal flap complications	Pain Corneal haze Slow recovery Higher rate of myopic regression Longer visual rehabilitation	[38,39,40,41,42]
LASEK (laser-assisted subepithelial keratomileusis)	A thin epithelial flap is created using alcohol, subepithelial ablation of the anterior stroma, and the epithelial flap is replaced	The cornea’s biomechanical stability is maintained Resistant to external impact No stromal flap complications	Pain for two days Blurry vision for several days	[17,43,44,45,46]
LASIK (laser-assisted in situ keratomileusis)	Creating a thin flap in the anterior cornea, posterior stroma ablation, and the flap is repositioned	Relatively fast recovery Less painful sensation Less corneal haze and maintenance of an intact Bowman’s membrane	Flap complications (dislocation, perforation), keratitis Epithelial ingrowth Keratectasia Weak to external impact Dry eye	[47,48,49,50,51]
FS-LASIK (femtosecond laser-assisted in situ keratomileusis)	Creating a thin corneal flap using a femtosecond laser, stroma ablation, and the flap is repositioned	More reproducible and predictable flap diameters	Flap-related complications Dry eye Price	[52,53,54,55,56]
SMILE (small-incision lenticule extraction)	Intrastromal lenticule extraction (a small corneal incision is made by femtosecond laser, and then the lenticule is removed mechanically)	No flap complications, the preservation of anterior stroma Very fast recovery	Price	[53,54,56,57,58,59,60]
Intraocular refractive surgery				
Phakic intraocular lens (pIOL) implantation	pIOL implantation into the anterior chamber (iris-fixated pIOL) Natural lens remains intact	Reversible procedure, Risk of developing cataracts is lower	Progressive corneal endothelial Cell loss Risk of traumatic iris-haptic de-enclavation Secondary glaucoma	[39,61,62,63,64,65]
Phakic intraocular lens (pIOL) implantation	pIOL implanted into the posterior chamber behind the iris Fixated in the ciliary sulcus, and the natural lens remains intact	Reversible procedure Quick visual rehabilitation Higher predictability Excellent optical outcomes	Cataract formation Progressive endothelial cell loss Pigment dispersion Secondary glaucoma	[66,67,68,69,70,71,72,73,74]
Refractive lens exchange (RLE) or clear lens extraction (CLE)	The removal of a natural lens with or without simultaneous insertion of a low-power or plano IOL	Relatively low cost	Late retinal detachment	[39,75,76,77,78]
